# Editorial: Sleep in children with rare disorders, volume II. Sleep disturbances in rare neurodevelopmental disorders: a call for deeper understanding

**DOI:** 10.3389/fneur.2025.1628150

**Published:** 2025-08-27

**Authors:** Karen Spruyt

**Affiliations:** INSERM, Université Paris Cité, Paris, France

**Keywords:** rare disorders, neurodevelopment, sleep disorder, wearables, sleep architecture

The complexities of sleep disturbances in rare disorders are gaining increasing attention. In this Research Topic, we explore the clinical, therapeutic, and neurobiological dimensions of four rare conditions: Anti-IgLON5 antibody-related encephalitis, Rett syndrome, Sotos syndrome, and Neurofibromatosis type 1. Each condition, while unique, shares a common feature—*disruptions in sleep*, which not only complicate patient care but also provide insights into the broader relationship between sleep, cognition, and neurodevelopment.

## Anti-IgLON5 antibody-related encephalitis: a rare but severe autoimmune disorder

Anti-IgLON5 antibody-related encephalitis is a rare and typically severe autoimmune disorder that predominantly affects middle-aged and elderly individuals. However, in this study by Feng et al., two pediatric cases, aged 2.5 and 9.6 years, were examined, providing a rare glimpse into the manifestation of the disease in children. Both patients presented with acute or subacute onset, marked by movement disorders, cognitive impairment, sleep disturbances, and psychiatric symptoms. Notably, the 9-year-old patient experienced profound sleep difficulties, with only 2 h of sleep per day—a striking indication of the severity of sleep dysfunction in this condition.

In the pediatric cohort, psychiatric symptoms, rapid onset, and stronger inflammatory responses were more pronounced compared to the adult population. Both patients received first- and second-line immunotherapies, though response rates varied. The study highlights that while immunotherapy can be effective to some extent, pediatric cases appear to require more tailored interventions. Moreover, the study underscores the unique nature of pediatric anti-IgLON5 antibody-related encephalitis, with sleep disturbances playing a critical role in early diagnosis and treatment outcomes.

## Rett syndrome: wearable monitoring of sleep and biovital parameters

Rett syndrome (RTT), a neurodevelopmental disorder primarily affecting females, is linked to mutations in the *MECP2* gene. RTT is associated with profound sleep disturbances, autonomic dysfunction, fatigue, and an increased risk of sudden death. In an innovative study (Leoncini et al.) testing the feasibility of continuous home monitoring, a wearable sensor was used to track heart rate, respiratory rate, and skin temperature in RTT patients. The study demonstrated that continuous 24-h monitoring is feasible, with data covering 77% of the total recording hours.

The results revealed significant findings regarding sleep disturbances in RTT. On average, patients had 9 h of sleep, but exhibited elevated heart rate variability (HRV) parameters, particularly during sleep. The HR/LF ratio, a marker of autonomic nervous system regulation, correlated with disease severity, sleep disturbances, hypoxia, and epileptic activity. These findings suggest that continuous monitoring using wearable sensors not only provides valuable clinical insights but also identifies potential biomarkers (such as HRmax% and HR/LF ratio) for fatigue and disease progression. This study opens new possibilities for patient management and treatment strategies in RTT.

## Sotos syndrome: sleep and neuropsychiatric correlates

Sotos syndrome (SoS) is a rare genetic disorder characterized by overgrowth and associated with mutations in the *NSD1* gene. While cognitive and behavioral impairments are prominent in affected individuals, especially those with microdeletions, sleep disturbances have not been thoroughly investigated. In this study (Frattale et al.), the prevalence of sleep disorders was assessed in a cohort of pediatric SoS patients, aiming to correlate sleep disturbances with neuropsychiatric profiles.

Preliminary findings suggest that sleep disturbances in SoS may exacerbate the neuropsychiatric symptoms commonly observed in these children. The study also highlights the importance of understanding the interplay between sleep, cognition, and behavior in SoS patients. This comprehensive neuropsychological evaluation provides a much-needed perspective on the potential relationship between sleep issues and the broader neurodevelopmental trajectory of children with Sotos syndrome.

## Neurofibromatosis type 1: sleep macrostructure in children

Neurofibromatosis type 1 (NF1) is a genetic condition that affects neurodevelopment and often leads to a range of cognitive and physical challenges. A study evaluating sleep macrostructure in children with NF1 found distinct sleep differences compared to typically developing controls (Carotenuto et al.). The study, which included 100 pre-pubertal children (50 with NF1 and 50 controls), assessed key sleep parameters through polysomnographic evaluations.

The findings were striking: children with NF1 exhibited reduced total sleep time (TST), lower sleep efficiency (SE%), and a decrease in N2 sleep percentage (*p* < 0.001). Conversely, these children showed increased awakenings, longer wake after sleep onset (WASO%), and more respiratory disturbances. These sleep disturbances suggest that NF1 may disrupt sleep architecture, potentially contributing to cognitive and behavioral issues commonly seen in these children. Notably, the study proposes that regular sleep assessment could be instrumental in diagnosing and managing both NF1 and related neurodevelopmental disorders, as addressing sleep disturbances could improve overall patient care and treatment outcomes.

## Implications and future directions

These studies underscore the complex relationship between rare neurodevelopmental and genetic disorders and sleep disturbances. From the severe cognitive and movement disorders seen in Anti-IgLON5 antibody-related encephalitis to the autonomic dysfunction and sleep issues of Rett syndrome, these disorders present unique challenges in clinical practice. However, each study also highlights the potential for innovative approaches in diagnosis and patient management, particularly through the use of wearable devices and continuous monitoring. Moreover, these findings provide important insights into the need for comprehensive neuropsychological and sleep assessments in children with rare disorders. By better understanding the role of sleep disturbances in these conditions, clinicians can develop more targeted and effective treatment strategies, improving the quality of life for affected individuals, and their families.

As research in this area continues to grow, the integration of advanced monitoring techniques and a deeper understanding of the neurobiological mechanisms underlying these disorders will be critical. Sleep, often viewed as a passive process, is emerging as a key player in the neurodevelopmental landscape, offering new avenues for therapeutic intervention and care. Addressing sleep disturbances in these conditions is crucial not only for improving the wellbeing of the patients but also for empowering families with restful nights.

Sleep research in rare diseases has progressed through the use of diverse methodologies—including longitudinal assessments, validation of home-monitoring devices, and the development of standardized sleep management protocols tailored to rare conditions. A recurring theme across these studies is the identification of autonomic dysregulation and disrupted sleep architecture as potential indicators of sleep-related pathophysiology. In parallel, wearable technologies have emerged as practical and scalable tools for capturing real-world sleep behaviors and physiological parameters, reinforcing the value of continuous, *in-situ* data collection in pediatric populations.

Despite these advances, the synthesis of findings into a cohesive framework for diagnosis, monitoring, and intervention remains in its early stages. Addressing this gap is critical to translate emerging evidence into effective and clinically actionable strategies for vulnerable pediatric populations. One step toward building such evidence is the systematic implementation of evidence-based reporting practices—such as using standardized checklists like the CARE (Case Report) guidelines—to enhance the rigor, transparency, and reproducibility of case reports and case series. Another key step involves routine screening for sleep disturbances in individuals with rare conditions, as well as in their caregivers and family members (1). Sleep disturbances represent critical clinical features that may function as predisposing, precipitating, or perpetuating factors in disease onset and progression. They can exacerbate existing comorbidities, modulate the trajectory of underlying conditions, or serve as early indicators of systemic physiological, psychological, or environmental dysregulation. The integration of standardized sleep assessments into routine clinical practice is therefore essential—not only for timely detection but also for guiding comprehensive, multidisciplinary management strategies aimed at improving outcomes and overall quality of life in vulnerable populations.

This Research Topic of studies serves as an important step forward in the ongoing effort to improve patient outcomes for children with rare and complex disorders. The need for interdisciplinary collaboration, sustained research, and patient-centered care has never been more apparent. These findings offer a beacon of hope for future advancements in both diagnostic and therapeutic strategies, guiding the way toward better care and improved lives for these vulnerable children and their families.
